# Effects of Diacetyl Flavoring Exposure in Mice Metabolism

**DOI:** 10.1155/2018/9875319

**Published:** 2018-06-28

**Authors:** Letícia Dias Lima Jedlicka, Juciara da Costa Silva, Aleksandro Martins Balbino, Giuseppe Bruno Neto, Danielle Zildeana Sousa Furtado, Heron Dominguez Torres da Silva, Fernanda de Barros Correia Cavalcanti, Karin Marie van der Heijden, Carlos Alberto Avellaneda Penatti, Etelvino José Henriques Bechara, Nilson Antonio Assunção

**Affiliations:** ^1^Instituto de Ciências Ambientais, Químicas e Farmacêuticas, Universidade Federal de São Paulo, Diadema, SP, Brazil; ^2^Instituto de Estudos em Saúde e Biológicas, Universidade Federal do Sul e Sudeste do Pará, Marabá, PA, Brazil; ^3^Universidade Nove de Julho, São Paulo, SP, Brazil; ^4^Instituto de Química, Universidade de São Paulo, SP, Brazil

## Abstract

Diacetyl is a flavoring that imparts a buttery flavor to foods, but the use or exposure to diacetyl has been related to some diseases. We investigated the effect of oral intake of diacetyl in male and female C57/Bl mice. We performed a target metabolomics assay using ultraperformance liquid chromatography paired with triple quadrupole mass spectrometry (UPLC-MS/MS) for the determination and quantification of plasmatic metabolites. We observed alterations in metabolites present in the urea and tricarboxylic acid (TCA) cycles. Peroxynitrite plasmatic levels were evaluated by a colorimetric method, final activity of superoxide dismutase (SOD) was evaluated by an enzymatic method, and mouse behavior was evaluated. Majority of the assay showed differences between control and treatment groups, as well as between genders. This may indicate the involvement of sex hormones in the regulation of a normal metabolic profile, and the implication of sex differences in metabolite disease response.

## 1. Introduction

It is increasingly common to use food additives to improve the appearance, quality, aroma, flavor, and longevity of food products [1]. Diacetyl is added to food products to improve taste by providing a butter-like flavor. Diacetyl (C_4_H_6_O_2_, cas number: 431-03-8) is a liquid at ambient temperature with a yellow or green-yellow color, and it is a diketone with a diffusive and pungent odor. Although diacetyl is directly added to human food and the compound is generally regarded as safe, other carbonyl compounds with low molecular weights can be toxic in large doses [2]. The acute oral LD_50_ (median lethal dose) of diacetyl for male and female rats is currently 3400 mg/kg and 3000 mg/kg, respectively, but the LD_50_ for mice has not yet been established [3].

Exposure to diacetyl has been extensively studied in recent years [4–11]. However, the majority of these studies focused on occupational exposure to diacetyl, mainly because of the occurrence of bronchiolitis obliterans in factory workers engaged in microwave popcorn production [5, 9].

We previously demonstrated the generation of acetyl radicals from the reaction between diacetyl and peroxynitrite [12–16] and also demonstrated the increase in protein acetylation* in vivo* after diacetyl consumption [17]. However, the broader effects of this flavoring compound have not been clearly studied. Even though foods with other chemical characteristics may result in highly reactive species once ingested, because diacetyl is an effective prooxidant, it will likely affect several metabolic routes [18, 19].

## 2. Materials and Methods

### 2.1. Experimental Protocol

Twelve-week-old male and female C57/Bl mice were divided into four groups (n = 6). The control groups did not receive any treatment, whereas treated groups received diacetyl in drinking water at concentrations of 300 mg/kg/day for 15 weeks. The mice were kept in a cabinet with 50–70% humidity at 19–23°C. A 12 h light/12 h dark cycle was used, and the mice were fed ad libitum. All procedures were approved by the UNIFESP Ethics Committee under number 975977013.

#### 2.1.1. Dosage Selection

Based on previous studies in our laboratory [20] and the study conducted by Colley et al. [3], we chose a dosage of 300 mg/kg/day of diacetyl diluted in drinking water. Daily administration was chosen because diacetyl is a food flavoring widely used in the food industry, and it is considered safe by several agencies [21–24].

#### 2.1.2. Determining Estrus Cycle Phase

The phase of the estrus cycle of the female mice was confirmed by vaginal smear. Before conducting the experiments and euthanasia in female mice, vaginal secretions were collected with a plastic pipette filled with 10 *μ*L of ultrapure water. The pipette tip was inserted into the mouse vagina to collect the secretion by aspiration. One drop of vaginal fluid was placed on a glass slide, dried at room temperature, and observed under a light microscope (Quimis, Brazil) with 10x and 40x objective lenses. Only female mice in the diestrus phase were used for the assay [25].

#### 2.1.3. Sample Collection and Preparation

The mice were euthanized in a CO_2_ gas chamber (RED Industry and Commerce, Brazil) with a flow rate of 30% of the chamber volume per minute. Blood was collected by cardiac puncture with heparinized syringes, transferred to microtubes, and then centrifuged at 5000 rpm for 1200 s at 4°C. Plasma was collected and a protease inhibitor cocktail (Calbiochem, Germany) was added following the manufacturer's instructions. Plasma was frozen in liquid nitrogen and then stored at -80°C.

### 2.2. Metabolomics Assay

After pooling the plasma samples from each experimental and control group, we detected a total of 33 metabolites using an AbsoluteIDQ™ p180 kit (Biocrates Life Sciences AG, Austria), following manufacturer's instructions: L-alanine (Ala), L-arginine (Arg), L-asparagine (Asn), L-aspartate (Asp), L-citrulline (Cit), L-glutamine (Gln), L-glutamate (Glu), L-glycine (Gly), L-histidine (His), L-isoleucine (Ile), L-leucine (Leu), L-lysine (Lys), L-methionine (Met), L-ornithine (Orn), L-phenylalanine (Phe), L-asymmetric dimethylarginine (ADMA), L-carnosine (Car), creatinine (Cre), L-DOPA (DOPA), L-histamine (Hst), L-kynurenine (Kyn), L-methionine sulphoxide (Met-So), L-proline (Pro), L-putrescine (Put), L-sarcosine (Sar), serotonin (5-HT), L-trans-4-hydroxyproline (t4-OH-Pro), L-taurine (Tau), L-threonine (Thr), L-tryptophan (Trp), L-tyrosine (Tyr), L-valine (Val), and acetyl ornithine (Ac-Orn).

Table 1 contains the standard parameters used in the data acquisition of metabolomics analysis, as well as the coefficients of determination, equations, retention time (t_R_), mass spectrometry (MS), tandem mass spectrometry (MS/MS), detection limit (LD), quantification limit (LQ), linear range, cone volume (CV), collision energy (V), and internal standards (IS).

#### 2.2.1. Statistical Analysis of Metabolomics Assay

Statistical analysis was conducted in two steps. Upon obtaining analyses of metabolites and performing the behavior screening, we used the software GraphPad Prism version 5.01 (San Diego, California, USA, https://www.graphpad.com/) to perform basic statistical comparisons of data with variance analysis through hypothesis testing (*t-test*), analysis of variance (ANOVA), and the post hoc Bonferroni test. Secondly, exploratory data analysis (EDA) was used to identify systematic relations between variables when there were either nonexistent or incomplete expectations as to the nature of those relations by hierarchical cluster analysis (HCA), by clustering tree or dendrogram and principal component analysis (PCA), or by linear dimensionality reduction.

The statistical analysis was performed using the data analysis software system Statistica, version 10.0, by StatSoft Inc. (2011). The first step enabled us to verify differences between paired data groups not matched by specific tests and ANOVA. The second step allowed the ranking and clustering of data, followed by analysis according to group.

#### 2.2.2. Enrichment Analysis of Metabolites

The enrichment analysis was performed using the global test package available at http://www.metaboanalyst.ca. It uses a generalized linear model to estimate a Q-statistic for each metabolite set, describing the correlation between compound concentration profiles and clinical outcomes.

### 2.3. SOD Assay

SOD was measured from plasma samples using the Superoxide Dismutase II Assay Kit (Cayman, USA). All reagents were used as supplied in the kit, prepared according to the manufacturer's instructions, and kept on ice during the assay. The absorbance was read at 450 nm using a plate reader. The SOD standard curve was plotted and linearized, and the SOD activity of the samples was calculated using the equation obtained from linear regression from the standards.

### 2.4. Peroxynitrite Assay

The serum peroxynitrite level was determined using the colorimetric method (Beckman et al., 1990). In brief, 10 *μ*L of serum was added to 90 *μ*L of cold NaOH (1.0 mol L^−1^) and mixed well. NaOH (1.0 mol L^−1^) was used as the blank. The blank absorbance was taken in a climatized quartz cuvette at 302 nm (10°C). A 10 *μ*L diluted sample was added to the cuvette and mixed three times. The absorbance at 302 nm of the diluted sample was recorded by a Spectramax spectrophotometer (Molecular Devices, USA).

### 2.5. Motor Locomotion and Behavior Testing

We investigated the chronic effect of diacetyl on animal motor locomotion and behavior. The open field test was chosen to determine the exploratory activity profile of the animal within the test arena, a divided area with central and peripheral (corner) limits. An animal spending time in the central area conveys low anxiety levels, whereas time spent in the corners conveys high anxiety levels [26]. The open field test arena consisted of an open square box with 50 cm sides, with a black square central area with 25 cm sides.

Upon 15 weeks of exposure to oral diacetyl, male and female mice were tested in the open field arena for a 300 s session. Behavior was analyzed and scored according to the total time spent locally, within either central or peripheral zones. In addition, we tabulated parameters of animal self-care and well-being (e.g., grooming, crossing, and standing up) as measures of normal social behavior. All behavioral tests were recorded with a Sony 12.1 camera and quantified using OPEN FLD software (Stefano Pupe, Brazil), which follows animal movement within the arena.

## 3. Results and Discussion

### 3.1. Metabolomic Differences in Male and Female Mice Treated Orally with 300 mg/kg/day of Diacetyl over 15 Weeks

Diacetyl is used as a food additive, but exposure and ingestion of this compound may lead to an imbalance in the body and the development of diseases [27, 28] which can alter the production of metabolic pathway intermediates. Thus, metabolites may reflect the conditions of the processes underlying cellular homeostasis, reflecting the relationships between cellular processes and biochemical pathways [28, 29]. The body tends to respond to these changes with intracellular and extracellular adjustments to maintain homeostasis [30, 31].

Sexual dimorphism is present in several species, in which nonsexual characteristics are present differently between the sexes. These differences encompass metabolites, lipid-derived molecules, cell regulatory processes, reactions to drugs [32], reactive oxygen species (ROS) signaling, and stress response [33]. The differing levels of hormones like progesterone, estrogen, and testosterone between the sexes are responsible for most of these differences. However, the differences go beyond the physical characteristics and may influence the functioning of the organism and may lead to differences in the metabolic profile [34, 35].

Because males and females may have different responses to a given treatment and/or susceptibility to disease, it is important to individually analyze the male and female results [19]. The metabolic analyses proceeded with male and female control groups in addition to groups treated with 300 mg/kg/day. Results were compared between the male and female controls in order to look for sex-dependent differences prior the analysis of the diacetyl treatment. We observed sex-specific differences in metabolite concentrations (Table 2).

**Figure 1:**
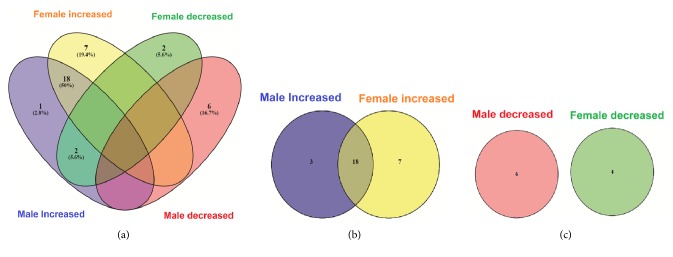
Changes in metabolic profile from male and female control groups and groups treated with 300 mg/kg/day of diacetyl. (a) Venn diagram of number of metabolites that increased and decreased in groups treated with diacetyl. (b) Venn diagram of number of metabolites that increased in groups treated with diacetyl. (c) Venn diagram of number of metabolites that decreased in groups treated with diacetyl.

We also observed differences in metabolite levels between sexes after diacetyl treatment (Figure 1(a)), which can be attributed to sexual dimorphism. The metabolites 5-HT, Ala, Arg, Asn, Cit, Gln, Gly, His, Ile, Leu, Met, Phe, Pro, Sar, Tau, Thr, Tyr, and Val increased in both male and female treated groups compared with the respective controls (Figure 1(b)). The metabolites Ac-Orn, ADMA, Car, Hist, Kyn, Met-So, and Trp increased in only the female treated group compared with its control group, whereas metabolites Glu, Put, and Orn increased in only the male treated groups compared to control. All these increased metabolites are involved in lipid and amino acid metabolism and genetic information processing pathways [36, 37]. All cellular functions depend on metabolism [38].

Different metabolites decreased in both groups treated with diacetyl in comparison with the respective control groups (Figure 1(c)). The metabolites Asp, T4-OH-Pro, Orn, and Glu showed decreased levels in the female treated group compared with the female control group; the metabolites Dopa, Car, Cre, Kyn, Trp, and Hst showed decreased levels in the male treated group. The other metabolites did not show a statistical difference compared with their respective controls.

**Figure 2:**
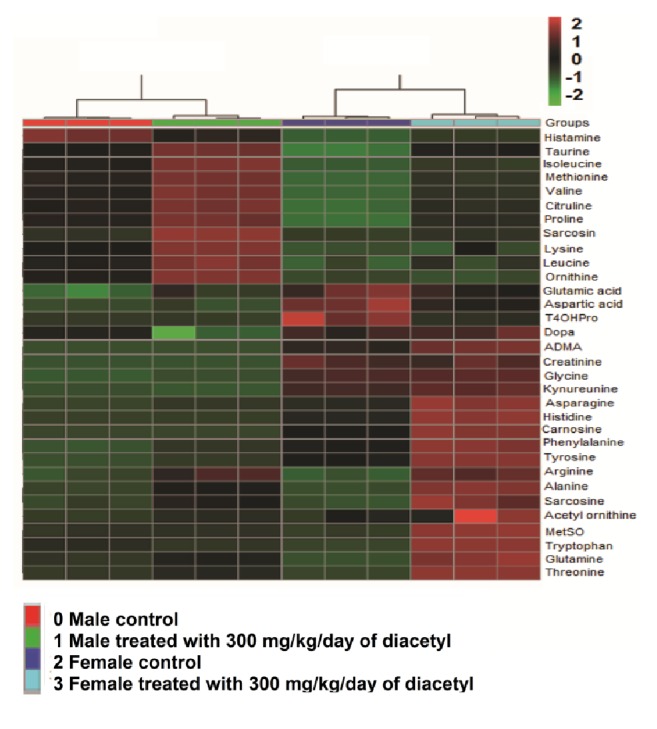
Heatmap of plasma metabolites from male controls, males treated with 300 mg/kg/day of diacetyl, female controls, and females treated with 300 mg/kg/day of diacetyl.

The plasma metabolites show different intensities in all groups (Figure 2). The metabolites with the highest values are in red, while the lowest appear in yellow and green, making it possible to visualize the difference of the metabolic profile among the groups with more clarity. The heatmap shows that diacetyl alters metabolism in both male and female groups. This data provides new insights into sex-specific responses to diacetyl treatment, as well as metabolism differences [39].

**Figure 3:**
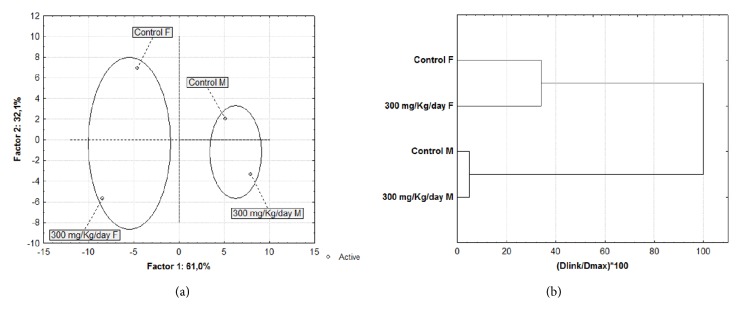
Multivariate analysis: principal component analysis (PCA) and dendrogram of male and female mice in control groups and groups treated with 300 mg/kg/day of diacetyl. (a) PCA of male and female mice in control groups and groups treated with 300 mg/kg/day of diacetyl. (b) Dendrogram showing Euclidean distance-based similarity of metabolite levels in control and treated groups.

The difference between male and female metabolic profiles after diacetyl treatment was associated with gender. The differences between the metabolic profiles of male and female groups is evident, but the response to diacetyl exposure was observed independently of sex. We performed multivariate analyses for all groups that included all analyzed metabolites (Figure 3). PCA analysis allowed us to identify differences or similarities between all groups. We observed an efficient separation between the male and female groups (Figure 3(a)). A subdivision separating the diacetyl-treated groups can also be seen, where control groups are in the upper quadrants and treated groups are in the lower quadrants.

The dendrogram provides more evidence for the separation of the four groups (Figure 3(b)). The* y*-axis represents the experimental groups in descending order of similarity, and the position of the line on the* x*-axis indicates the distances between the groups. The female groups have the greatest similarity because they have the shortest distance between them (Figure 3(b)). The female groups formed the first branch; the male groups formed the second branch. The male and female branches remained distinct because of the differences between their respective metabolic profiles.

The first branch has two subbranches that distinguish female controls from females treated with 300 mg/kg/day of diacetyl. The second branch also contained two subbranches that distinguished male controls from treated males. These results show both the dissimilarity between sexes and the difference in treatment with 300 mg/kg/day of diacetyl. This difference in metabolic profile found between male and female groups may be related to natural sexual dimorphism.

Sex hormones also contribute to the differences between sexes and are associated with incidence and progression of some diseases [20–22]. For example, estrogens are atheroprotective and vasoprotective and may exert potent antioxidant actions [21]. Estrogen has an antiapoptotic effect in muscle and neural tissues upon stress. Testosterone also shows antiapoptotic activity in muscle cell lines upon oxidative stress, but testosterone in rat myocytes showed a proapoptotic effect [38]. Metabolic profile differences between male and female control groups are likely attributable to the effects of estrogen.

### 3.2. Possible Pathways and Diseases Altered in Males and Females from Groups Treated with 300 mg/kg/day of Diacetyl

When an organism's homeostasis is disrupted, this may lead to some dysfunction that impacts metabolism or promotes disease. The organism can also develop a stress response to help to maintain homeostasis [39, 40]. Diacetyl and other *α*-dicarbonyl compounds promote chemical disorders including mutagenesis, cancer, aging, diabetes, neurodegenerative processes, several metabolism errors, and inflammatory diseases [13].

To relate the diacetyl intake status with the plasma metabolite expression data, we performed the metabolite set enrichment analysis with a database (http://metaboanalyst.ca). The metabolites were ranked according to the increases or decreases in groups treated with 300 mg/kg/day of diacetyl when compared with the respective controls. This analysis showed metabolic pathways that were altered after diacetyl treatment in male and female groups.

The metabolic profile reflects the physiological, developmental, and pathological processes of the organism, which can inform disease prognosis [41]. In this way, metabolomic analysis can be a valuable tool to analyze pathway alterations, which helps understand biochemical disease mechanisms and the effects of drugs and potentially toxic substances [42].

**Figure 4:**
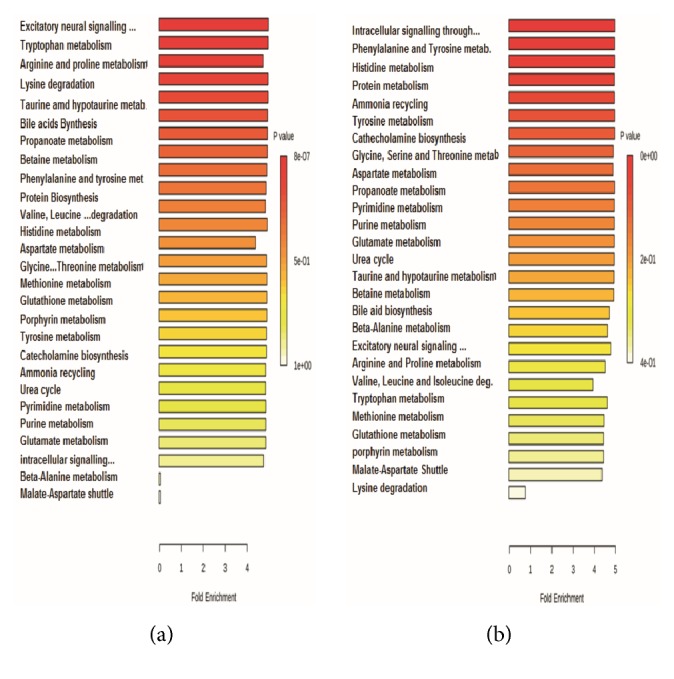
Quantitative set enrichment analysis of metabolites in male and female groups treated with 300 mg/kg/day of diacetyl. (a) Affected pathways in males. (b) Affected pathways in females.

The increased metabolites observed in male and female groups treated with 300 mg/kg/day of diacetyl may be involved in some major pathways (Figures 4(a) and 4(b)). The metabolite set enrichment analysis confirmed involvement of diacetyl treatment in some pathways. The table data set generated by quantitative analysis using the MetaboAnalyst tools is available in the supplementary material (SM1 and SM2). The same pathways were affected in males and females treated with diacetyl, but each group responded somewhat differently. The treated males had a greater change in excitatory neural signaling through 5-HT, whereas the treated females showed a greater change in intracellular signaling through histamine H2 receptor and histamine.

**Figure 5:**
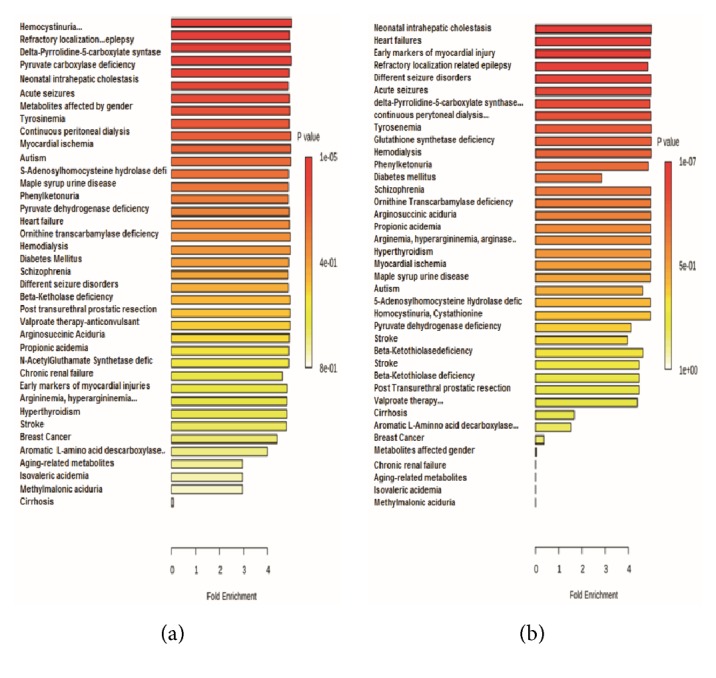
Quantitative set enrichment analysis of metabolites in male and female groups treated with 300 mg/kg/day of diacetyl. (a) Related diseases for male treated group. (b) Related diseases for female treated group.

Some diseases affect the metabolic profile, so a precise metabolite quantification assists in proper diagnosis [40]. Metabolites were ranked according to their increases or decreases in groups treated with 300 mg/kg/day of diacetyl compared with the respective control group (SM 3, SM 4). Metabolite expression is correlated with the likelihood of certain diseases (Figures 5(a) and 5(b)). We included diseases if at least five of the investigated metabolites involved in their pathological mechanisms were altered after diacetyl treatment. The same diseases affected both male and female groups, but the most relevant male disease was homocystinuria and the most relevant female disease was intrahepatic cholestasis (Figures 5(a) and 5(b)).

### 3.3. Behavior Screening in Female and Male Mice after Long-Term Exposure to Diacetyl

**Figure 6:**
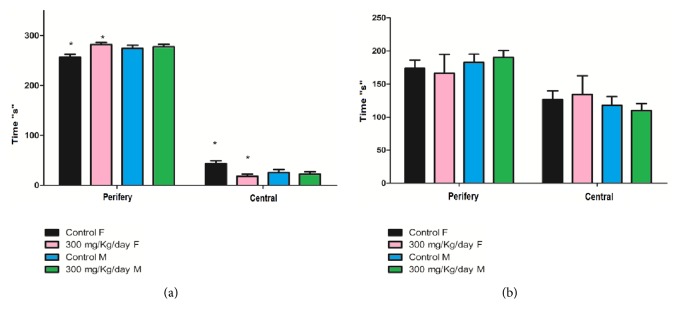
Open field test results, with time (s) spent in periphery and central regions. (a) First round of analysis. (b) Second round of analysis.

Behavior screening was performed to investigate the effects of diacetyl intake on mice behavior. We observed a discrete difference between male and female control groups, as well as between the female control group and the female group treated with diacetyl (Figure 6(a)). These differences can be associated with differing endocrine regulation due to sexual dimorphism [43–49]. A second round of open field experiments with other groups of animals that received the same diacetyl dose confirmed the same altered behavior (Figure 6(b)).

Other parameters were also observed in the open field test, including grooming, stand-up events, and crossing frequency between the periphery and central regions (Table 3). However, we did not observe changes in the first or second rounds of analysis.

### 3.4. SOD and Peroxynitrite Assay

Peroxynitrite and other reactive nitrogen species help regulate some physiological functions. However, the generation of peroxynitrite can happen in inflammation and antioxidant system failures [29].

**Figure 7:**
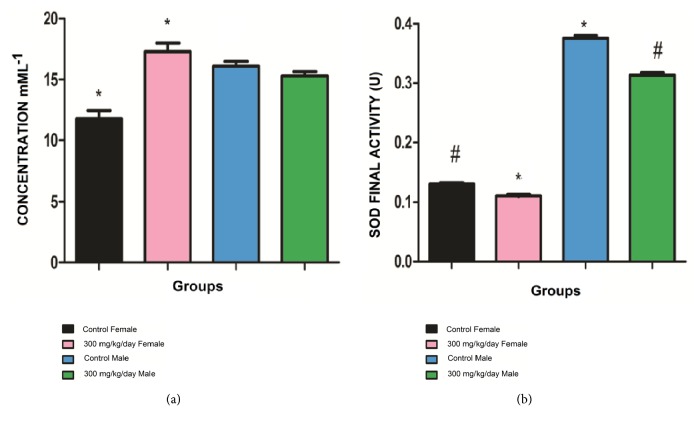
Peroxynitrite levels and SOD final activity in male and female control groups and groups treated with 300 mg/kg/day of diacetyl. (a) Peroxynitrite levels in male and female control groups and groups treated with 300 mg/kg/day of diacetyl. (b) SOD final activity in male and female control groups and groups treated with 300 mg/kg/day of diacetyl. *∗* groups with statistical difference (ANOVA and Posttest Bonferroni) from female control group (p < 0.0001). Values expressed as mean ± SEM. # groups with statistical difference (ANOVA and Posttest Bonferroni) from male control groups (p < 0.0001). Values expressed as mean ± SEM.

We observed different responses to diacetyl between males and females regarding peroxynitrite levels in mML^−1^ (Figure 7(a)) and SOD final activity (Figure 7(b)). Peroxynitrite levels were higher in treated females (17.25 ± 0.71) than in control females (11.76 ± 0.68) (Figure 7(a)). Peroxynitrite levels were similar between the control males (16.08 ± 0.39) and the treated males (15.29 ± 0.34) (Figure 7(a)). These results demonstrate that female groups produced more peroxynitrite when exposed to 300 mg/kg/day of diacetyl, while male groups did not exhibit the same increase. Peroxynitrite is a prooxidant substance and is involved in oxidative stress [28, 50, 51]. These data suggest there is a sex-related difference in response during oxidative stress. Testosterone stimulates the formation of peroxynitrite and peroxynitrite precursors such as superoxide, nitric oxide, and xanthine oxidase [34], whereas estradiol is associated with decreased peroxynitrite levels [52]. The differing responses to diacetyl exposure may be because sex hormones naturally induce higher baseline peroxynitrite levels in male controls than in female controls.

Superoxide dismutase (SOD) is an antioxidant enzyme that defends the body against oxidative stress [52]. SOD final activity (U) decreased in treated male and female groups (0.313 ± 0.004 and 0.111 ± 0.001, respectively) when compared to the male and female control groups (0.375 ± 0.004 and 0.130 ± 0.001, respectively) (Figure 7(b)). This suggests diacetyl decreases SOD activity.

Diacetyl treatment increased plasma peroxynitrite levels and decreased SOD final activity in female groups when compared to control. SOD final activity also decreased in the male treated group when compared to control, but peroxynitrite levels did not change. Both female and male groups treated with diacetyl demonstrated the imbalance between the prooxidants and antioxidants, suggesting that diacetyl intake is negatively correlated with oxidative stress.

## 4. Conclusion 

Metabolomics of male and female C57/black mice showed different biochemical patterns. Diacetyl administered orally to male and female mice promoted metabolomic changes in a sex-dependent manner. This suggests the involvement of sex hormones in the regulation of a normal metabolic profile, and the implication of sex-based differences in metabolite disease response.

The metabolite levels of 5-HT, Ala, Arg, Asn, Cit, Gln, Gly, His, Ile, Leu, Met, Phe, Pro, Sar, Tau, Thr, Tyr, and Val were increased in male and female treated groups. Some metabolites increased only in the female treated group compared to control (Ac-Orn, ADMA, Hst, Kyn, Met-SO, and Trp), and some metabolites increased only in the male treated group compared to control (Glu, Orn, and Put). The metabolite levels of Asp, Glu, Orn, and T4-OH-Pro decreased in the female treated group compared to control; the metabolite levels of Cre, Dopa, Hst, Kyn, Car, and Trp decreased in the male treated group compared to control.

The current work confirms the studies that demonstrated the diacetyl can modify peptides and proteins by acetylation [8, 10], and various metabolic routes involve acetylation, including the urea cycle [47]. Most of the proteins that participate in these routes become acetylated, which indicates a potential role of acetylation in the regulation of cell metabolism.

Diacetyl intake may cause changes in protein structure, likely due to uncontrolled acetylation [8]. Altered protein structure can lead to increased ROS levels and misfolded or degraded proteins. Protein degradation may facilitate an increase in circulating amino acids and affects protein turnover [53]. Protein turnover may cause increased ammonia levels due to protein catabolism [31]. Ammonia is a toxic substance, of nitrogen metabolism, and one of the ways of eliminating excess ammonia occurs through the urea cycle. Upregulation of the urea cycle increases ureagenesis, a process that may cause changes in hepatic nitrogen homeostasis and hormonal regulation [54]. This mechanism would explain the increased metabolites related to protein biosynthesis and amino acid metabolism, both of which increase substrates of the urea cycle.

The open field test showed discrete differences between motor locomotion and social behavior expressed through anxiety/fear when comparing male and female control groups and groups treated with diacetyl. Females are less vulnerable to stress than males [55], but they are more affected by repeated stress than males [56]. Such behavioral changes may be related to changes in metabolic profile. For example, the metabolite 5-HT increased in both treated groups and is involved in the pathophysiology of anxiety disorders [57]. The alteration of 5-HT also promotes changes in behavioral expression [57]. Aside from the serotonergic system, the adrenergic and glutaminergic systems are also involved in anxiety and behavior changes.

Some metabolite alterations are found in patients or animals with hepatic or renal injury, which demonstrates the possible toxicity of diacetyl intake. However, most metabolite alterations are associated with diseases related to increase of oxidative stress and inflammation. SOD activity decreased in both treated groups while peroxynitrite increased in only female treated groups, demonstrating that there is an imbalance between prooxidants and antioxidants in diacetyl-treated animals. Our results suggest that long-term intake of diacetyl is associated with oxidative stress, a relationship previously demonstrated by our group through* in vitro *studies. [12–15]

## Figures and Tables

**Table 1:** Coefficients of determination, equations, retention time (t_R_), MS, MS/MS, LD, LQ, linear range, CV, V, and IS.

**Metabolites**	**R** ^**2**^	**Equation**	**t**_**R**_ **∗**	**MS**	**MS/MS**	**LD** **∗** **∗**	**LQ** **∗** **∗**	**Linear range** **∗** **∗**	**CV**	**V**	**IS**
**5-HT**	1	y = 0.015x- 0.020	1.27	241	60	0.42	1.40	5.00-400.00	28	18	Serotonin D_4_
**Ala**	1	y = 0.007x - 0.037	1.62	225	44	0.05	0.18	20.00 - 600.00	22	10	Orn D_6_
**Ac-orn**	0.99	y = 30.165x - 10.120	1.12	310	217	0.01	0.06	0.50 - 40.00	32	24	Orn D_6_
**ADMA**	0.92	y = 0.0168x +-0.001	1.29	338	46	0.07	0.20	0.25 - 20.00	22	14	Ala D_4_
**Arg**	0.99	y = 0.015x + 0.048	1.12	310	217	0.03	0.10	5.00 - 400.00	34	14	Arg ^15^N_2_
**Asn**	1	y = 0.0149x + 0.024	1.18	268	87	0.01	0.05	5.00 - 400.00	20	16	Asn ^15^N_2_
**Asp**	0.99	y = 0.010x - 0.987	1.41	269	116	0.01	0.03	5.00 - 400.00	24	14	Asp D_3_
**Car**	1	y = 0.010x + 0.001	1.00	362	136	0.08	0.26	0.50 - 40.00	24	34	His ^13^C_6_
**Cit**	1	y = 0.021x + 0.007	1.33	311	113	1.13	3.78	5.00 - 400.00	22	24	Cit ^13^C D_4_
**Cre**	0.99	y = 0.008x + 0.041	0.16	114	44	0.72	2.42	10.00 - 800.00	32	14	Creatinine D_3_
**Dopa**	0.99	y = 0.218x - 0.085	1.33	333	198	0.16	0.55	0.50 - 40.00	26	12	Dopa D_3_
**Gln**	1	y = 0.019x + 0.006	1.22	282	130	0.03	0.12	20.00-1600.00	22	16	Gln D_5_
**Glu**	0.99	y = 0.012x + 0.030	1.42	283	130	1.29	4.30	10.00 - 800.00	26	16	Glu D_3_
**Gly**	1	y = 0.002x + 0.004	1.29	211	76	0.10	0.35	25.00- 2000.00	22	10	Gly ^13^C2 ^15^N
**His**	1	y = 0.018x + 0.004	1.00	291	110	0.76	2.55	5.00 - 400.00	22	22	His ^13^C_6_
**Hst**	0.97	y= 0.110x - 0.078	1.01	247	154	0.03	0.11	5.00 - 400.00	22	12	His ^13^C_6_
**Ile**	1	y = 0.001x - 0.001	2.51	267	69	0.15	0.52	5.00 - 400.00	24	28	Ile ^13^C_6_
**Kyn**	0.99	y = 0.024x + 0.004	2.43	344	146	0.08	0.27	1.00 - 80.00	22	24	Tyr D_4_
**Leu**	0.99	y = 2.542 e^− 005^x + 2.791 e^– 006^	2.51	267	43	0.06	0.02	5.00 - 400.00	24	38	Ile ^13^C_6_
**Lys**	0.99	y = 0.037x - 0.512	2.50	417	324	0.07	0.26	10.00 - 800.00	26	12	Orn D_6_
**Met**	1	y = 0.017x - 0.002	2.14	285	104	0.21	0.73	5.00 - 400.00	22	18	Met D_3_
**Met-So **	0.98	y = 0.025x + 0.0435	1.34	301	88	0.01	0.05	1.00 - 80.00	18	30	Met D_3_
**Orn**	0.98	y = 0.046x - 0.212	2.38	403	310	0.002	0.01	5.00 - 400.00	24	12	Orn D_6_
**Phe**	0.99	y = 0.015x + 0.068	2.56	301	120	0.006	0.02	5.00 - 400.00	24	20	Phe D_5_
**Pro**	0.99	y = 0.012x + 0.105	1.61	251	70	0.04	0.14	5.00 - 400.00	26	20	Pro D_7_
**Put**	0.97	y=-0.033x +0.270	2.55	266	114	0,94	3.15	0.10 - 8.00	26	12	Putrescine D_4_
**Sar**	1	y= 2.68x - 0.157	1.62	225	90	0.03	0.10	1.00 - 80.00	20	10	Sarcosine D_3_
**T4-OH-Pro**	0.99	y= 0.009x - 0.005	1.29	267	132	0.01	0.06	1.00 - 80.00	24	14	Pro D_7_
**Tau**	0.91	y= 0.016 x + 0.035	1.00	261	126	0.03	0.10	2.50 - 200.00	30	14	Taurine ^13^C_2_
**Thr**	1	y = 0.020x + 0.020	1.53	255	73	0.07	0.26	5.00 - 400.00	22	18	Thr^13^C4 ^15^N
**Trp**	1	y = 0.015x - 0.015798	2.48	317	136	0.01	0.05	5.00 - 400.00	26	16	Trp ^15^N_2_
**Tyr**	1	y= 0.0258201x +0.0219755	1.94	340	188	0.003	0.01	5.00 - 400.00	26	22	Tyr D_4_
**Val**	0.99	y = 0.013x + 0.021	2.21	2534	72	0.01	0.06	10.00 - 800.00	24	16	Val D_8_

**∗** **min,** **∗** **∗** **(** **µ** **mol L** ^-**1**^ **)**

**Table 2:** Plasma metabolite concentrations from female and male groups treated with 300 mg/kg/day of diacetyl for 15 weeks.

**Metabolite**	** Control Female** **∗**	**300 mg/kg/day Female** **∗** **∗**	**Control Male** **∗**	**300 mg/kg/day Male** **∗** **∗**
**5-HT**	0.87 ± 0.03	1.33 ± 0.03 (↑)	1.27 ± 0.03	6.83 ± 0.03 (↑)
**Ac-Orn**	4.37 ± 0.70	11.00 ± 3.10 (↑)	2.97 ± 0.03	4.13 ± 0.03 (*↔*)
**ADMA**	63.00 ± 2.00	104.00 ± 2.35 (↑)	0.6 ± 0.11	0.57 ± 0.03 (*↔*)
**ALA**	215.00± 4.23	706.00 ± 5.78 (↑)	240.15 ± 4.20	421,00 ± 3.72 (↑)
**Arg**	70.00 ± 1.47	122.00 ± 1.54 (↑)	75.00 ± 1.86	114.00 ± 3.47 (↑)
**Asn**	110.00 ± 4.25	449.0 ± 12.08 (↑)	30 ± 0.81	65.0 ± 1.46 (↑)
**Asp**	197.00 ± 13.90	97.00 ± 12.77 (↓)	16.00 ± 1.90	18.00 ± 2.70 (*↔*)
**Car**	51.0 0± 0.73	146.00 ± 3.02 (↑)	0.97 ± 0.03	0.70 ± 0.06 (↑)
**Cit**	6.83 ± 0.68	26.00 ± 0.63 (↑)	40.00 ± 0.94	66.00 ± 0.70 (↑)
**Cre**	8667.00 ± 654.00	8642.00 ± 7370.00 (*↔*)	19.00 ± 1.01	8.20 ±11.00 (↓)
**Dopa**	0.87 ± 0.03	0.93 ± 0.03 (*↔*)	0.70 ± 0.00	0.43 ± 0.30 (↓)
**Gln**	239.00 ± 6.56	1898.00 ± 61.40 (↑)	485.00 ± 15.40	760.00 ± 15.10 (↑)
**Glu**	136.00 ± 8.50	111.00 ± 5.40 (↓)	62.00 ± 3.44	93.00 ± 10.50 (↑)
**Gly**	439.00 ± 15.60	1988.00 ± 11.00 (↑)	56.00 ± 4.07	82.00 ± 3.09 (↑)
**His**	439.00 ± 2.96	1988.00 ± 27.40 (↑)	56.00 ± 0.20	82.00 ± 1.31 (↑)
**Hst**	0.30 ± 0.00	0.50 ± 0.00 (↑)	1.43 ± 0.03	0.97 ± 0.03 (↓)
**Ile**	19.00 ± 0.20	36.00 ± 1.15 (↑)	66.00 ± 0.79	114.00 ± 0.55 (↑)
**Kyn**	22.00 ± 0.30	27.00 ± 0.52 (↑)	2.07.0 ± 0.10	0.8 ± 0.00 (↓)
**Leu**	33.00 ± 9.87	63.00 ± 11.70 (↑)	113.0 ± 9.17	213.00 ± 4.50 (↑)
**Lys**	73.00 ± 3.69	106.00 ± 38.00 (↑)	182.00 ± 4.05	348.0 ± 1.07 (↑)
**Met**	6.80 ± 0.06	18.00 ± 0.55 (↑)	33.00 ± 0.72	50.00 ± 0.35 (↑)
**Met-SO**	0.80 ± 0.26	26.00 ± 0.20 (↑)	2.57 ± 0.24	2.77 ± 0.12 (*↔*)
**Orn**	17.00 ± 1.74	11.00 ± 2.33 (↓)	60.0 ± 0.17	120.00 ± 0.68 (↑)
**Phe**	118.00 ± 1.23	203.00 ± 2.59 (↑)	71.00 ± 0.95	93.00 ± 0.19 (↑)
**Pro**	12.00 ± 0.22	46.00 ± 0.72 (↑)	69.00 ± 0.26	108.00 ± 2.13 (↑)
**Put**	6.07 ± 0.52	7.05 ± 0.37 (*↔*)	0.73 ± 0.07	1.13 ± 0.03 (↓)
**Sar**	12.00 ± 0.48	41.00 ± 2.29 (↑)	14.00 ± 0.76	26.00 ± 0.96 (↑)
**T4-OH-Pro**	14.00± 1.41	1.87 ± 0.22 (↓)	0.87 ± 0.07	0.5 ± 0.32 (*↔*)
**Tau**	58.00 ± 4.33	186.00 ± 3.50 (↑)	208.00 ± 1.70	329.00 ± 2.89 (↑)
**Thr**	75.00 ± 0.92	382.00 ± 0.41 (↑)	101.00 ± 2.14	138.00 ± 2.89 (↑)
**Trp**	69.00 ± 0.43	192.00 ± 0.58 (↑)	90.00 ± 0.20	81.00 ± 0.58 (↓)
**Tyr**	148.00 ± 3.04	297.00 ± 0.21 (↑)	69.00 ± 1.79	96.0 ± 1.41 (↑)
**Val**	**47.00 **±** 0.22**	**99.00 **±** 1.35 (**↑**)**	**155.00 **±** 2.44**	**240.00 **±** 2.90 (**↑**)**

↑ significant increase in concentration compared to respective control group; ↓ significant decrease in concentration compared to respective control group; *↔* concentration does not have significant difference from control group.

**Table 3:** Open field test in male and female control groups and groups treated with 300 mg/kg/day of diacetyl.

**Group**	**Round of analysis**	**Crossing events**	**Stand-up events**	**Grooming events**
Females, control	1	33.60 ± 1.47	37.40 ± 2.94	2.40 ± 2.07
Females, 300 mg/kg/day diacetyl treatment	1	23.20 ± 5.59	29.20 ± 2.37	3.60 ± 1.89
Males, control	1	24.33 ± 1.92	28.33 ± 1.93	1.50 ± 0.50
Males, 300 mg/kg/day diacetyl treatment	1	24.33 ± 3.56	21.50 ± 1.52	3.17 ± 0.95
Females, control	2	40.00 ±6.85	31.00 ± 9.14	2.00 ± 0.45
**Females, 300 mg/kg/day diacetyl treatment**	2	39.60 ± 7.52	31.60 ± 6.87	3.80 ± 0.92
Males, control	2	37.60 ± 3.34	20.40 ± 5.77	2.60 ± 0.93
**Males, 300 mg/kg/day diacetyl treatment**	2	42.00 ±2.59	19.40 ± 2.01	2.40 ± 0.25

## Data Availability

Data are available in the Supplementary Materials.

## Supplementary Materials

Supplementary 1 Supplementary Material 1. Affected pathways in male groups. This material included the metabolite set for probable pathways, the total number of metabolites, expected value, P value, and the false discovery rate (FDR), in male groups.

Supplementary 2 Supplementary Material 2. Affected pathways in female groups. This material included the metabolite set for probable pathway, the total number of metabolites, expected value, P value, and the false discovery rate (FDR), in female groups.

Supplementary 3 Supplementary Material 3. Probably disease in male groups. This material included the metabolite set for probable disease, the total number of metabolites, expected value, P value, and the false discovery rate (FDR), in male groups.

Supplementary 4 Supplementary Material 4: Probably disease in female groups. This material included the metabolite set for probable desease, the total number of metabolites, expected value, P value, and the false discovery rate (FDR), in female groups.
