# Supplementary material for: Effects of Diacetyl Flavoring Exposure in Mice Metabolism
Source: Biomed Res Int. 2018 Jun 28;2018:9875319. doi: 10.1155/2018/9875319 (PMC6051334; doi:10.1155/2018/9875319)
Supplement: Supplementary 1 — Supplementary Material 1. Affected pathways in male groups. This material included the metabolite set for probable pathways, the total number of metabolites, expected value, P value, and the false discovery rate (FDR), in male groups. [file 9875319.f1.docx]

SM 1: Affected pathways in male groups

| **Metabolite Set** | **Total (metabolites)** | **Expected** | **P value** | **FDR** |
| --- | --- | --- | --- | --- |
| Excitatory neural signalling through 5-htr 4 and serotonin \| excitatory neural signalling through 5-htr 7 and serotonin \| excitatory neural signalling through 5-htr 6 and serotonin | 5 | 20.0 | 0,000308 | 0,008327 |
| Tryptophan metabolism | 34 | 20.0 | 0,002457 | 0,033166 |
| Arginine and proline metabolism | 26 | 20.0 | 0,004229 | 0,038063 |
| Lysine degradation | 13 | 20.0 | 0,024175 | 0,16318 |
| Taurine and hypotaurine metabolism | 7 | 20.0 | 0,035662 | 0,19257 |
| Bile acid biosynthesis | 49 | 20.0 | 0,004487 | 0,20192 |
| Propanoate metabolism | 18 | 20.0 | 0,23981 | 0,83459 |
| Betaine metabolism | 10 | 20.0 | 0,25318 | 0,83459 |
| Phenylalanine and tyrosine metabolism | 13 | 20.0 | 0,29346 | 0,83459 |
| Protein biosynthesis | 19 | 20.0 | 0,30911 | 0,83459 |
| Valine, leucine and isoleucine degradation | 36 | 20.0 | 0,36258 | 0,84381 |
| Histidine metabolism | 11 | 20.0 | 0,37503 | 0,84381 |
| Aspartate metabolism | 12 | 20.0 | 0,42966 | 0,89238 |
| Glycine, serine and threonine metabolism | 26 | 20.0 | 0,53284 | 1,0276 |
| Methionine metabolism | 24 | 20.0 | 0,60088 | 1,0512 |
| Glutathione metabolism | 10 | 20.0 | 0,66189 | 1,0512 |
| Porphyrin metabolism | 22 | 20.0 | 0,66189 | 1,0512 |
| Tyrosine metabolism | 38 | 20.0 | 1,1796 | 1,6762 |
| Catecholamine biosynthesis | 5 | 20.0 | 1,1796 | 1,6762 |
| Ammonia recycling | 18 | 20.0 | 1,4601 | 1,9712 |
| Urea cycle | 20 | 20.0 | 0,1557 | 2,0018 |
| Pyrimidine metabolism | 36 | 20.0 | 2,1765 | 2,4485 |
| Purine metabolism | 45 | 20.0 | 2,1765 | 2,4485 |
| Glutamate metabolism | 18 | 20.0 | 2,1765 | 2,4485 |
| Intracellular signalling through histamine h2 receptor and histamine | 5 | 20.0 | 5,8441 | 6,3116 |
| Beta-alanine metabolism | 13 | 20.0 | 0.82788 | 0.82924 |
| Malate-aspartate shuttle | 8 | 20.0 | 0.82924 | 0.82924 |
