# Supplementary material for: Effects of Diacetyl Flavoring Exposure in Mice Metabolism
Source: Biomed Res Int. 2018 Jun 28;2018:9875319. doi: 10.1155/2018/9875319 (PMC6051334; doi:10.1155/2018/9875319)
Supplement: Supplementary 2 — Supplementary Material 2. Affected pathways in female groups. This material included the metabolite set for probable pathway, the total number of metabolites, expected value, P value, and the false discovery rate (FDR), in female groups. [file 9875319.f2.docx]

SM 2: Affected pathways in female groups

| **Metabolite set** | **Total (metabolites)** | **Expected** | **P value** | **FDR** |
| --- | --- | --- | --- | --- |
| Intracellular signalling through histamine h2 receptor and histamine | 5 | 20.0 | 0 | 0 |
| Phenylalanine and tyrosine metabolism | 13 | 20.0 | 0,007952 | 0,048805 |
| Histidine metabolism | 11 | 20.0 | 0,00862 | 0,048805 |
| Protein biosynthesis | 19 | 20.0 | 0,011375 | 0,048805 |
| Ammonia recycling | 18 | 20.0 | 0,012668 | 0,048805 |
| Tyrosine metabolism | 38 | 20.0 | 0,013356 | 0,048805 |
| Catecholamine biosynthesis | 5 | 20.0 | 0,013356 | 0,048805 |
| Glycine, serine and threonine metabolism | 26 | 20.0 | 0,014461 | 0,048805 |
| Aspartate metabolism | 12 | 20.0 | 0,023337 | 0,007001 |
| Propanoate metabolism | 18 | 20.0 | 0,028762 | 0,077658 |
| Pyrimidine metabolism | 36 | 20.0 | 0,11422 | 0,22706 |
| Purine metabolism | 45 | 20.0 | 0,11422 | 0,22706 |
| Glutamate metabolism | 18 | 20.0 | 0,11422 | 0,22706 |
| Urea cycle | 20 | 20.0 | 0,11774 | 0,22706 |
| Taurine and hypotaurine metabolism | 7 | 20.0 | 0,21017 | 0,03783 |
| Betaine metabolism | 10 | 20.0 | 0,34431 | 0,58103 |
| Bile acid biosynthesis | 49 | 20.0 | 3,4895 | 5,5421 |
| Beta-alanine metabolism | 13 | 20.0 | 4,1517 | 6,2275 |
| Excitatory neural signalling through 5-htr 4 and serotonin \| excitatory neural signalling through 5-htr 7 and serotonin \| excitatory neural signalling through 5-htr 6 and serotonin | 5 | 20.0 | 5,8441 | 8,3048 |
| Arginine and proline metabolism | 26 | 20.0 | 0.0017573 | 0.0023723 |
| Valine, leucine and isoleucine degradation | 36 | 20.0 | 0.001983 | 0.002468 |
| Tryptophan metabolism | 34 | 20.0 | 0.002011 | 0.002468 |
| Methionine metabolism | 24 | 20.0 | 0.0038582 | 0.0045292 |
| Glutathione metabolism | 10 | 20.0 | 0.0047359 | 0.0051148 |
| Porphyrin metabolism | 22 | 20.0 | 0.0047359 | 0.0051148 |
| Malate-aspartate shuttle | 8 | 20.0 | 0.0060054 | 0.0062363 |
| Lysine degradation | 13 | 20.0 | 0.43871 | 0.43871 |
