# Supplementary material for: Effects of Diacetyl Flavoring Exposure in Mice Metabolism
Source: Biomed Res Int. 2018 Jun 28;2018:9875319. doi: 10.1155/2018/9875319 (PMC6051334; doi:10.1155/2018/9875319)
Supplement: Supplementary 3 — Supplementary Material 3. Probably disease in male groups. This material included the metabolite set for probable disease, the total number of metabolites, expected value, P value, and the false discovery rate (FDR), in male groups. [file 9875319.f3.docx]

SM 3: Probably disease in male groups

| **Metabolite set** | **Total** | **Expected** | **P value** | **Fdr** |
| --- | --- | --- | --- | --- |
| Homocystinuria, cystathionine beta-synthase deficiency | 5 | 20.0 | 0,003232 | 0,005946 |
| Refractory localization-related epilepsy | 10 | 20.0 | 0,00375 | 0,005946 |
| Delta-pyrrolidine-5-carboxylate synthase deficiency | 5 | 20.0 | 0,004574 | 0,005946 |
| Pyruvate carboxylase deficiency | 10 | 20.0 | 0,021973 | 0,21424 |
| Neonatal intrahepatic cholestasis | 12 | 20.0 | 0,057966 | 0,45213 |
| Acute seizures | 14 | 20.0 | 0,13951 | 0,78338 |
| Metabolites affected by gender | 9 | 20.0 | 0,17112 | 0,78338 |
| Tyrosinemia i | 5 | 20.0 | 0,17638 | 0,78338 |
| Continuous ambulatory peritoneal dialysis (capd) | 15 | 20.0 | 0,01985 | 0,78338 |
| Myocardial ischemia | 6 | 20.0 | 0,02309 | 0,78338 |
| Autism | 8 | 20.0 | 0,25318 | 0,78338 |
| S-adenosylhomocysteine hydrolase deficiency | 5 | 20.0 | 0,25318 | 0,78338 |
| Maple syrup urine disease | 9 | 20.0 | 0,02782 | 0,78338 |
| Phenylketonuria | 7 | 20.0 | 0,28121 | 0,78338 |
| Pyruvate dehydrogenase deficiency (e3) | 7 | 20.0 | 0,36258 | 0,94271 |
| Heart failure | 10 | 20.0 | 0,04044 | 0,98574 |
| Ornithine transcarbamylase deficiency (otc) | 10 | 20.0 | 0,54296 | 1,0325 |
| Glutathione synthetase deficiency | 8 | 20.0 | 0,54896 | 1,0325 |
| Hemodialysis | 14 | 20.0 | 0,55227 | 1,0325 |
| Diabetes mellitus (mody), non-insulin-dependent | 19 | 20.0 | 0,58511 | 1,0325 |
| Schizophrenia | 26 | 20.0 | 0,62487 | 1,0325 |
| Different seizure disorders | 24 | 20.0 | 0,63874 | 1,0325 |
| Beta-ketothiolase deficiency | 8 | 20.0 | 0,66189 | 1,0325 |
| Post transurethral prostatic resection | 5 | 20.0 | 0,66189 | 1,0325 |
| Valproate therapy: anticonvulasant hypersensitivity syndrome valproate associated hepatotoxicity | 5 | 20.0 | 0,66189 | 1,0325 |
| Argininosuccinic aciduria (asl) | 6 | 20.0 | 0,78312 | 1,1747 |
| Propionic acidemia | 8 | 20.0 | 1,6648 | 2,3721 |
| N-acetylglutamate synthetase deficiency. Nags deficiency | 5 | 20.0 | 1,7031 | 2,3721 |
| Chronic renal failure | 13 | 20.0 | 2,1389 | 2,8765 |
| Early markers of myocardial injury | 14 | 20.0 | 3,5369 | 0,4598 |
| Argininemia. Hyperargininemia, arginase deficiency | 5 | 20.0 | 5,4685 | 6,6647 |
| Hyperthyroidism | 5 | 20.0 | 5,4685 | 6,6647 |
| Stroke | 5 | 20.0 | 6,8523 | 8,0982 |
| Breast cancer | 5 | 20.0 | 0.0058207 | 0.0066767 |
| Aromatic l-amino acid decarboxylase deficiency | 12 | 20.0 | 0.01613 | 0.017974 |
| Aging-related metabolites | 6 | 20.0 | 0.074428 | 0.076387 |
| Isovaleric acidemia | 9 | 20.0 | 0.074428 | 0.076387 |
| Methylmalonic aciduria (mma) | 8 | 20.0 | 0.074428 | 0.076387 |
| Cirrhosis | 23 | 20.0 | 0.82924 | 0.82924 |
