# Supplementary material for: Effects of Diacetyl Flavoring Exposure in Mice Metabolism
Source: Biomed Res Int. 2018 Jun 28;2018:9875319. doi: 10.1155/2018/9875319 (PMC6051334; doi:10.1155/2018/9875319)
Supplement: Supplementary 4 — Supplementary Material 4: Probably disease in female groups. This material included the metabolite set for probable desease, the total number of metabolites, expected value, P value, and the false discovery rate (FDR), in female groups. [file 9875319.f4.docx]

SM 4: Probably disease in female groups

| **Metabolite set** | **Total** | **Expected** | **P value** | **Fdr** |
| --- | --- | --- | --- | --- |
| Neonatal intrahepatic cholestasis | 12 | 20.0 | 3,58e-05 | 0,001396 |
| Heart failure | 10 | 20.0 | 0,000134 | 0,002614 |
| Early markers of myocardial injury | 14 | 20.0 | 0,000912 | 0,001185 |
| Refractory localization-related epilepsy | 10 | 20.0 | 0,003048 | 0,029716 |
| Different seizure disorders | 24 | 20.0 | 0,011529 | 0,057023 |
| Acute seizures | 14 | 20.0 | 0,011692 | 0,057023 |
| Delta-pyrrolidine-5-carboxylate synthase deficiency | 5 | 20.0 | 0,011968 | 0,057023 |
| Continuous ambulatory peritoneal dialysis (capd) | 15 | 20.0 | 0,012514 | 0,057023 |
| Tyrosinemia i | 5 | 20.0 | 0,013159 | 0,057023 |
| Glutathione synthetase deficiency | 8 | 20.0 | 0,015798 | 0,061611 |
| Hemodialysis | 14 | 20.0 | 0,018699 | 0,066296 |
| Phenylketonuria | 7 | 20.0 | 0,030384 | 0,098748 |
| Diabetes mellitus (mody), non-insulin-dependent | 19 | 20.0 | 0,046253 | 0,13876 |
| Schizophrenia | 26 | 20.0 | 0,080728 | 0,22489 |
| Ornithine transcarbamylase deficiency (otc) | 10 | 20.0 | 0,11691 | 0,26116 |
| N-acetylglutamate synthetase deficiency. Nags deficiency | 5 | 20.0 | 0,11766 | 0,26116 |
| Argininosuccinic aciduria (asl) | 6 | 20.0 | 0,11782 | 0,26116 |
| Propionic acidemia | 8 | 20.0 | 0,12054 | 0,26116 |
| Argininemia. Hyperargininemia, arginase deficiency | 5 | 20.0 | 0,16615 | 3.24e-5 |
| Hyperthyroidism | 5 | 20.0 | 0,16615 | 3.24e-5 |
| Myocardial ischemia | 6 | 20.0 | 0,31225 | 0,55951 |
| Maple syrup urine disease | 9 | 20.0 | 0,32692 | 0,55951 |
| Autism | 8 | 20.0 | 0,34431 | 0,55951 |
| S-adenosylhomocysteine hydrolase deficiency | 5 | 20.0 | 0,34431 | 0,55951 |
| Homocystinuria, cystathionine beta-synthase deficiency | 5 | 20.0 | 6,8518 | 0.0010689 |
| Pyruvate dehydrogenase deficiency (e3) | 7 | 20.0 | 0.001983 | 0.0029746 |
| Stroke | 5 | 20.0 | 0.0021145 | 0.0030543 |
| Beta-ketothiolase deficiency | 8 | 20.0 | 0.0047359 | 0.0061567 |
| Post transurethral prostatic resection | 5 | 20.0 | 0.0047359 | 0.0061567 |
| Valproate therapy: anticonvulasant hypersensitivity syndrome valproate associated hepatotoxicity | 5 | 20.0 | 0.0047359 | 0.0061567 |
| Cirrhosis | 23 | 20.0 | 0.0060054 | 0.0075551 |
| Aromatic l-amino acid decarboxylase deficiency | 12 | 20.0 | 0.2302 | 0.28056 |
| Pyruvate carboxylase deficiency | 10 | 20.0 | 0.23836 | 0.2817 |
| Breast cancer | 5 | 20.0 | 0.60858 | 0.69808 |
| Metabolites affected by gender | 9 | 20.0 | 0.89659 | 0.98131 |
| Chronic renal failure | 13 | 20.0 | 0.96133 | 0.98131 |
| Aging-related metabolites | 6 | 20.0 | 0.98131 | 0.98131 |
| Isovaleric acidemia | 9 | 20.0 | 0.98131 | 0.98131 |
| Methylmalonic aciduria (mma) | 8 | 20.0 | 0.98131 | 0.98131 |
